# Image analysis in medical imaging: recent advances in selected examples

**DOI:** 10.2349/biij.6.3.e32

**Published:** 2010-07-01

**Authors:** G Dougherty

**Affiliations:** Applied Physics and Biomedical Imaging, California State University Channel Islands, California, United States of America

**Keywords:** Mammography, osteoporosis, tortuosity, scoliosis, osteoarthritis

## Abstract

Medical imaging has developed into one of the most important fields within scientific imaging due to the rapid and continuing progress in computerised medical image visualisation and advances in analysis methods and computer-aided diagnosis. Several research applications are selected to illustrate the advances in image analysis algorithms and visualisation. Recent results, including previously unpublished data, are presented to illustrate the challenges and ongoing developments.

## INTRODUCTION

A multitude of diagnostic medical imaging modalities are used to probe the human body. Interpretation of the resulting images requires sophisticated image processing methods that enhance visual interpretation, and image analysis methods that provide automated or semi-automated tissue detection, measurement and characterisation. In general, multiple transformations will be needed in order to extract the data of interest from an image, and a hierarchy in the processing steps will be evident, e.g. enhancement will precede restoration, which will precede analysis, feature extraction and classification. Often these are performed sequentially, but more sophisticated tasks will require feedback of parameters back to preceding steps so that the processing includes a number of iterative loops.

Several ongoing areas of research have been selected to highlight novel developments in analysis and display, in the hope that the methodologies may be transferred to other applications.

## SELECTED APPLICATIONS

### Mammography

Mammography is the single most important technique in the investigation of breast cancer, the most common malignancy in women. It can detect disease at an early stage when therapy or surgery is most effective. However, the interpretation of screening mammograms is a repetitive task involving subtle signs, and suffers from a high rate of false negatives (10%–30% [1, 2]), and false positives (10–20% [3, 4]). Computer-aided diagnosis (CAD) aims to increase the predictive value of the technique by pre-reading mammograms to indicate the locations of suspicious abnormalities and analyse their characteristics, as an aid to the radiologist.

**Figure 1:**
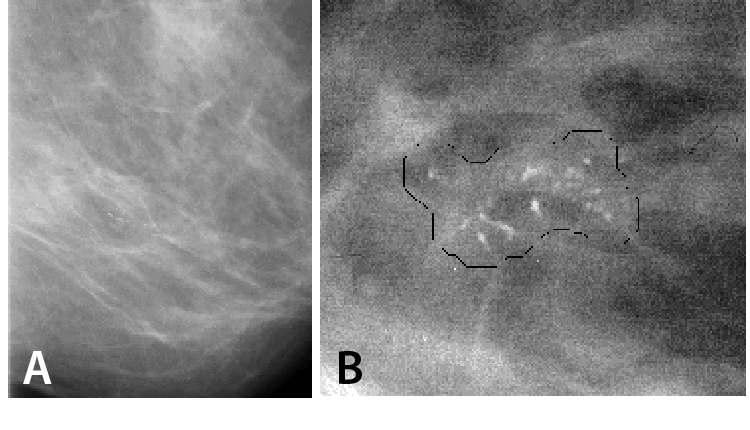
(a) A mammogram showing a cluster of microcalcifications and (b) computer-estimated margin around a cluster of microcalcifications.

**Figure 2:**
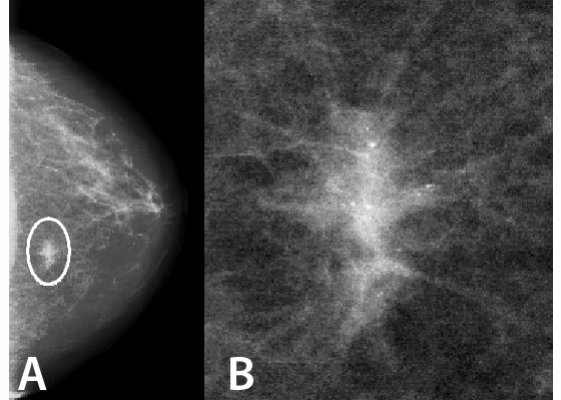
(a) A mammogram showing a stellate lesion and (b) a magnified image of the lesion.

About 90% of breast cancers arise in the cells lining the milk ducts of the breast, and are known as *ductal carcinoma in situ* (DCIS). Once the tumour extends beyond the lining of the ducts it is termed invasive, and can metastasise to other sites in the body. Radiographic indications fall mainly into two categories, microcalcifications and lesions (masses) (Figure 1). Microcalcifications are the primary means of detecting *in situ* carcinomas (i.e. those within the milk ducts); they are typically in the order of several hundred microns or smaller in diameter, and tend to occur in clusters. Most lesions are ill-defined in shape, often with tissue strands or spiculations radiating out from them, and similar in radio-opacity to the surrounding normal tissue (Figure 2). The imaging requirements in mammography are stringent, both in terms of spatial and contrast resolution.

CAD performance and reliability depends on a number of factors including the optimisation of lesion segmentation, feature selection, reference database size, computational efficiency, and the relationship between the clinical relevance and the visual similarity of the CAD results. Segmentation of the breast region serves to limit the search area for lesions and microcalcifications. It is also useful to adjust the grey values of the image to compensate for varying tissue thickness; one way to do this is to add grey values according to the *Euclidian distance map*, mapping distances to the skin line in a smoothed version of the mammogram [5]. Noise in the image can be reduced by median filtering, although this can disturb the shape and/or contrast of small structures. An improved technique [6] combines the results of morphological erosion and dilation using multiple structuring elements.

To improve the accuracy and reliability of mass region segmentation, a large number of computing algorithms have been proposed, developed and tested, including multi-layer topographic region growth algorithms [7, 8, 9], active contour (snake) modeling [10], adaptive region growth [11], a radial gradient index (RGI)-based modeling [12], and a dynamic programming-based boundary tracing (DPBT) algorithm [13]. Due to the diversity of breast masses and overlap of breast tissue in the 2-D projected images as well as the limited testing datasets, it is very difficult to compare the performance and robustness of these segmentation methods [14].

**Figure 3:**
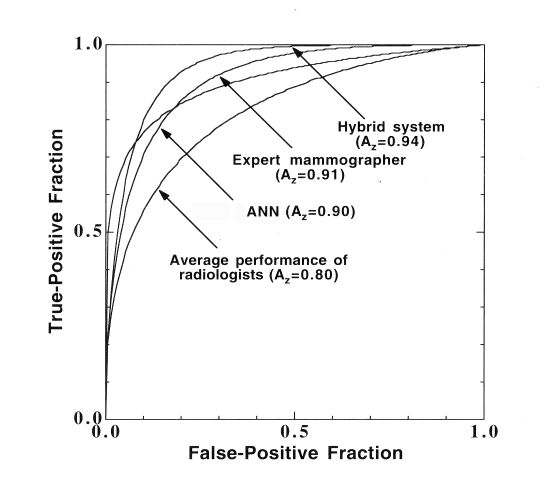
Receiver operating characteristic (ROC) curves illustrating the performances of a computer classification method and radiologists in the task of distinguishing between malignant and benign lesions. ANN indicates an artificial neural network using cumulative edge gradient features, and the hybrid system using several features. (Reprinted from [32], with permission from Elsevier).

Features which are useful for characterising lesions include their degree of spiculation, shape and texture [15]. Spiculation features commonly involve the calculation of image gradient using, for example, the Sobel masks [16]. The cumulative edge gradient, from the Sobel magnitude-of-edges image, can be plotted as a histogram of the radial angle, from the Sobel phase-of-edges image, to determine the degree of spiculation [17, 18]. The FWHM (full width at half maximum) of the gradient is able to distinguish spiculated masses from smooth masses. Others have used multi-scale oriented line detectors to detect and measure spiculated masses [19]. The centres of mass lesions tend to be circular so that specific filters can be used [20]. The boundary of the lesion can be unwrapped, and its difference from a smoothed version used to characterise the degree of spiculation [21]. Other relevant features include asymmetry, which would include automatic registration of left and right breast images [22], and changes with time [23]. Wavelets and Gabor filters have been extensively investigated and compared [24], and Gabor filters have performed better and corresponded well to the human vision (in particular for the sensitivity of edge detection) [25]. Other popular texture features derived from the co-occurrence matrices [26] and Fourier transformation [27] have also been tested. Recently, fractal dimension has been shown to be an effective and efficient metric for assessing texture in the detection and classification of suspicious breast mass regions [28]. Fractal dimension can be used to distinguish between malignant and benign breast masses [29], and has a high correlation with visual similarity [30, 31]. Since fractal dimension is a feature computed in the frequency domain, it has the advantage of being invariant to the lesion position and to rotation and scale. Most researchers extract several features and use principal component analysis to identify the most successful combinations. Different methods can be evaluated by receiver operating characteristic (ROC) analysis (Figure 3), but cannot be compared with each other unless the same image databases were used.

Microcalcifications can be described by the morphology (shape, area, brightness *etc.*) of individual calcifications, and the spatial distribution and heterogeneity of individual calcifications within a cluster. They can be enhanced by thresholding the image, and morphologically opening it using a structuring element to eliminate very small objects while preserving the size and shape of the calcifications [33]. Isolated calcifications have little clinical significance, so many investigators have incorporated a clustering algorithm into the classification system, in which only clusters that contain more than a selected number of microcalcifications within a region of chosen size are retained [34]. Such schemes are easily implemented using the k-nearest-neighbour (k-NN) algorithm. Both spatial distribution and heterogeneity of the features within a cluster can be used to qualitatively correlate with a radiologist’s criterion, and a classifier such as a neural network is used to estimate the likelihood of malignancy [35, 36]. Bayesian methods [37], discriminant analysis [38], rule-based methods [39] and genetic algorithms [40] have also been used in classification.

Computer assisted diagnosis (CAD) systems do not have to be perfect since they are used with a radiologist and not alone. Since the cost of a missed cancer is much greater than the misclassification of benign findings, they should be developed to reduce false negatives (*i.e.*, have a high sensitivity) even at the cost of some acceptable number of false positives (*i.e.*, reasonable specificity).

### Bone strength and osteoporosis

**Figure 4:**
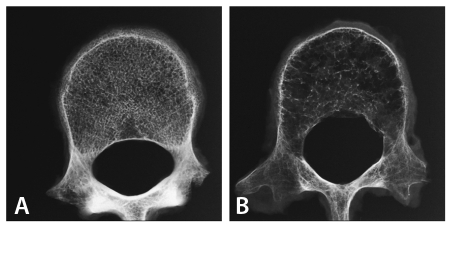
Radiographic images of lumbar vertebrae from (a) normal and (b) osteoporotic patients.

Osteoporosis is a prevalent bone disease characterised by a loss of bone strength and consequent fracture risk. Because it tends to be asymptomatic until fractures occur, relatively few people are diagnosed in time for effective therapy to be administered. Clinically, bone mineral density, BMD, is widely used to diagnose and assess osteoporosis. Changes in bone mass are commonly used as a surrogate for fracture risk. Although bone mineral density, BMD, is widely used clinically, it has been increasingly realised that internal bone architecture is also an important determinant of the mechanical strength of bone and can lead to an earlier and more accurate diagnosis of osteoporosis [41–44]. Figure 4 shows how the loss of trabeculae in osteoporosis results in a less well-connected, and therefore weaker, structure. The limited resolution of commercial CT scanners precludes proper resolution of the trabecular structure; however, CT images retain some of this architectural information [45, 46], albeit degraded by the inadequate modulation transfer function (MTF) of the imaging system, and this can be characterised by the fractal signature of the trabecular bone (viz. its fractal dimension as a function of spatial frequency [47, 48] and its lacunarity [49], a measure of the distribution of gaps in an image).

Fractal dimension describes how an object occupies space and is related to the complexity of its structure. Fractal dimension is related to the radial Fourier power spectrum of an image as a consequence of using fractional Brownian motion as a model for natural fractals. Estimates of the fractal signature, which are independent of the CT scanner used and its settings, can be obtained by correcting the power spectrum for image degradation due to noise, and for image blurring by the modulation transfer function (MTF) of the scanner [50]. However, changes in fractal dimension need to be interpreted with care. Global fractal dimension does not change monotonically with decalcification (Table 1).

**Table 1:** A visual classification scheme for the assessment of the trabecular structure used for the determination of the degree of osteoporosis.

**Class**	**Bone strength**	**Spongiosa pattern**	**Marrow size**	**Fractal dimension of ROI**
1. Healthy	High	Homogeneously dense with granular structure	Small, homogeneous	Low, fractal
2. Beginning demineralisation	Normal	Discrete disseminated intertrabecular areas	Medium, inhomogeneous	High, multi-fractal
3. Osteopaenia	Low	Confluent intertrabecular areas less than 50% of the cross-sectional surface	Large, inhomogeneous	High, multi-fractal
4. Severe osteoporosis	Very low	Confluent intertrabecular areas more than 50% of the cross-sectional surface.	Very large, homogeneous	Low, multi-fractal

Lacunarity measures the distribution of gap sizes in data: the greater the heterogeneity the greater the lacunarity. An efficient algorithm for estimating lacunarity analyses deviations from translational invariance of an image’s brightness distribution using gliding-box sampling [49]. Lacunarity can be defined in terms of the local first and second moments, measured for each neighbourhood size, about every pixel in the image, i.e.

(1) $$\text{L(}r)=1+\{\mathrm{var}(r)/{\text{mean}}^{2}(r)\}\}$$

where mean(*r*) and var(*r*) are the mean and variance of the pixel values, respectively, for a neighbourhood size *r*. An average lacunarity value can be calculated across the scale range of the bone image to indicate the average gap (marrow) size and its degree of heterogeneity.

In a pilot study, we measured three features which have been used as surrogates for bone quality (bone mineral density, the average fractal dimension and the average lacunarity) from CT scans of sixteen patients whose bone strength had been previously assessed [51]. The data is shown in Table 2.

**Table 2:** Features measured from CT scans, and ground-truth class from bone strength.

**Patient**	**BMD (mg cm^-3^)**	**Average fractal dimension**	**Average Lacunarity**	**Class**
A	204	2.5	1.3	1
B	205	2.55	1.35	1
C	186.2	2.45	1.4	1
D	174.1	2.48	1.42	1
E	165.6	2.69	1.4	2
F	150.2	2.71	1.45	2
G	135.9	2.74	1.42	2
H	159.8	2.65	1.46	2
I	127.9	2.65	1.54	3
J	98.1	2.68	1.5	3
K	138.1	2.7	1.55	3
L	125.5	2.72	1.54	3
M	81.7	2.58	1.36	4
N	103.3	2.57	1.3	4
O	101.8	2.55	1.32	4
P	110.4	2.53	1.28	4

**Figure 5:**
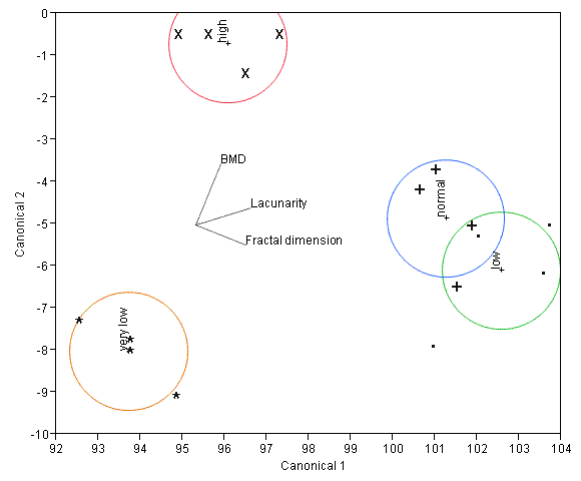
Canonical plot of surrogates for bone strength. Ground truth conditions are indicated by separate symbols. The directions of the three features are shown in the canonical space by the labelled rays. The size of each circle corresponds to a 95% confidence limit for the mean (marked with a +) of that group.

Linear discriminant analysis showed that the patients could be correctly classified into four classes (identified in table 1) according to bone strength on the basis of canonicals which are linear combinations of these three features (Figure 5), but misclassification rates of 12.5% occurred if only BMD and one other feature was used. These features, when used together, are potentially useful in monitoring bone strength and predicting future fracture risk using CT or MRI images.

### Tortuosity

The clinical recognition of elevated tortuosity or integrated curvature is important in the diagnosis of many diseases. Increased vascular tortuosity, for example, affects the flow haemodynamics and can lead to aneurysm (rupture of the blood vessels), and the tortuosity of retinal blood vessels can be an early indicator of systemic diseases.

**Figure 6:**
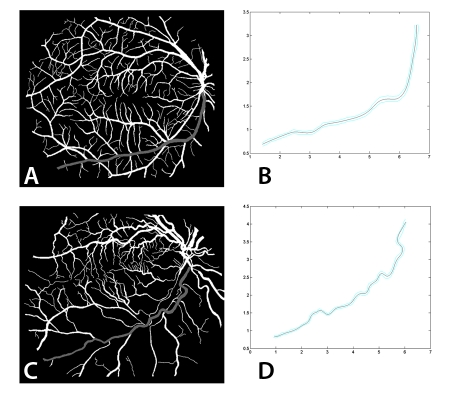
Binarised retinal image and the smoothest path through a selected vessel for a normal patient, (a) and (b), and a patient showing retinal pigmentosa, (c) and (d). (The axes in (b) and (d) are in relative units).

Vessel tortuosity does not have a formal clinical definition but a tortuosity metric should be additive and invariant to affine transformations of a vessel (translation, rotation and scaling) [52–54] if it is to correlate with the qualitative assessment of an expert observer. One metric is the cumulative angle moved as an observer passed along the mid-line data points of the vessel, divided by the length of the vessel (which we shall refer to as *M*). Another metric is based on the root-mean-square curvature of a unit speed curve, obtained by an approximating polynomial spline fitting to the mid-line data points [55, 56]. The fitted curve is not required to pass through each point, but rather approach it to within a distance related to the radius of the local vessel, and is the smoothest path under these circumstances; it is not restricted to the discrete pixel grid so that it can more closely correspond to the actual vessel (Figure 6). Approximating polynomial spline fitting captures the essential tortuosity of the vessels without having to place undue reliance on the accuracy of each extracted mid-line point, or employ arbitrary smoothing methods. Again this root-mean-square value would be divided by the length of the curve to give the tortuosity metric (which we shall refer to as *K*). Although these metrics are not strictly dimensionless, they are often referred to the length of the vessel in pixels and their units omitted: they can then be treated as numbers which rank the tortuosity of vessels obtained under the same imaging conditions.

These analyses are construed directly in three dimensions (3-D) and their clinical validity has been established [57] using clinical data sets from computed tomography angiography, CTA, and magnetic resonance angiography, MRA.

**Figure 7:**
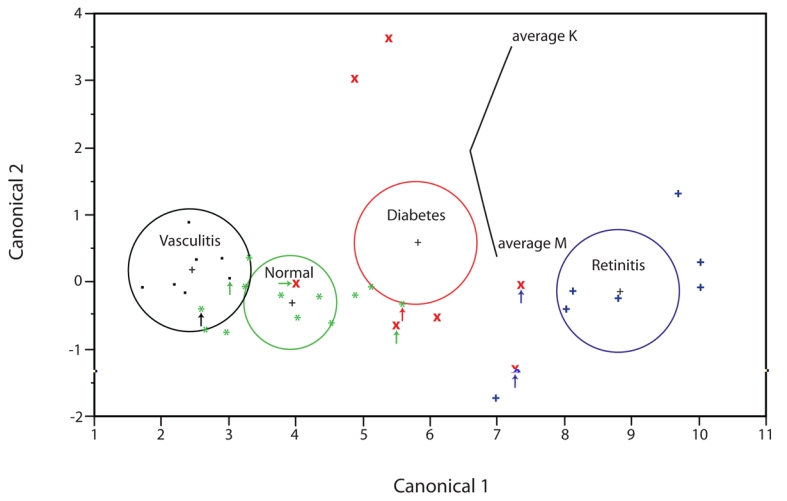
Canonical plot of data from 330 retinal vessels. Data from the ground truth conditions are indicated by separate symbols, each indicating the mean of 10 measurements. The directions of the features, M and K, are shown in the canonical space by the labeled rays. The size of each circle corresponds to a 95% confidence limit for the mean (marked with +) of that group; groups with significantly different values of tortuosity have non-intersecting circles. The small arrows indicate misclassified data points.

These tortuosity metrics are able to distinguish between normal vessels and some retinal pathologies in retinal fundus images [58, 59], with a high positive predictive value, PPV (Table 3). Discriminant analysis shows that the two metrics can be used together for classifying vessels into the four classes based on their tortuosity (Figure 7). However, the misclassification rate is 21% using prior probabilities proportional to their occurrence. A more successful approach would be to use the tortuosity metrics together as a test for a single condition in referred patients already suspected of being at risk. Other features are relevant for particular pathologies, e.g. the number of aneurysms and extent of haemorrhaging and exudate in diabetic retinopathy [60, 61].

**Table 3:** Parameters characterising the tests for the three pathologies using the tortuosity metrics, *M* and *K*, whose values for the normals were 4.88 ± 1.17 and 7.74 ± 2.01, respectively. A total of 330 vessels were measured. PPV (NPV) is the positive (negative) predictive value. Results are calculated using prevalences in the general population, and using the probabilities of each abnormal condition in the sample (viz. 7/19), in parentheses.

**Tortuosity metric**		**Retinitis pigmentosa**	**Diabetic retinopathy**	**Vasculitis**
*M*	Mean ± sd	10.67 ± 1.10	6.69 ± 2.04	2.85 ± 0.50
	sensitivity	0.807 (0.993)	0.207 (0.520)	NA (0.917)
	specificity	1.000 (0.996)	0.999 (0.930)	NA (0.875)
	PPV	0.924 (0.993)	0.805 (0.812)	NA (0.811)
	NPV	1.000 (0.996)	0.978 (0.769)	NA (0.948)
*K*	Mean ± sd	17.62 ± 2.72	11.91 ± 2.22	4.99 ± 0.94
	sensitivity	0.705 (0.973)	0.263 (0.752)	NA (0.858)
	specificity	1.000 (0.990)	0.997 (0.907)	NA (0.809)
	PPV	0.924 (0.982)	0.733 (0.824)	NA (0.724)
	NPV	1.000 (0.984)	0.979 (0.862)	NA (0.907)

### Scoliosis

Scoliosis is a complicated condition characterised by a lateral curvature of the spine and accompanied by rotation of the vertebrae on its axis [62]. Despite the risks associated with repeated exposure to ionising radiation [63], radiography remains the most accurate method of assessing the scoliotic curvature. A scoliotic angle, determined from an erect antero-posterior (AP) radiograph of the full spine, is routinely used to clinically characterise the curvature. There are variations in the definition of the scoliotic angle and the methodology for measuring it [64]. Notwithstanding the differences, the methodologies identify the vertebrae at the upper and lower limits of the curve and, in some methods, the apical vertebrae (i.e. the most laterally deviated), and manually measure angles between defining points or lines within them.

Due to the errors associated with manual Cobb angle measurement from plain radiographs, a number of authors have developed computer-assisted techniques using digitised radiographs [65–69]. Several of these studies [65–67] report lower variability with computer-based techniques, but one [69] found no improvement in computer-based over manual measurement. However, all of these reported techniques require manual selection of features by the user, thus introducing inter and intra-observer measurement error. To the author’s knowledge, no existing technique allows completely automated measurement of spinal curvature.

The tortuosity metrics (*M *and *K*) can be used to characterise the curvature of the spine in patients with idiopathic scoliosis by iteratively fitting piece-wise polynomial splines to the geometric centres of the vertebrae as seen in 2-D A-P radiographs [70]. The values were strongly correlated with the Cobb and Ferguson scoliotic angles (Table 4). The tortuosity metrics use the positions of all the affected vertebrae, rather than just two or three select vertebrae, and produce a single measure of tortuosity for each patient even when a mixture of curvatures is present. The direct use of positional data removes the vagaries of defining end points and determining the appropriate lines to draw through variably-shaped endplates.

**Table 4:** The correlation matrix, showing the (Pearson) correlation coefficients and their 95% confidence intervals.

	**Cobb**	**Ferguson**	***M***	***K***
Cobb	1	0.994 [0.985,0.997]	0.862 [0.679, 0.944]	0.866 [0.730, 0.954]
Ferguson	0.994 [0.985,0.997]	1	0.850 [0.654, 0.939]	0.873 [0.702, 948]
*M*	0.862 [0.679, 0.944]	0.850 [0.654, 0.939]	1	0.996 [0.990, 0.998]
*K*	0.866 [0.730, 0.954]	0.873 [0.702, 948]	0.996 [0.990, 0.998]	1

Since spinal curvature occurs in three-dimensions, it would be preferable to acquire three-dimensional images of the spine. It has been shown that reformatted coronal slices produced from transverse CT slices of supine patients and combined into a single image by a z-projection can be used to measure scoliotic angles [71]. The variability in angle measurements is similar, but the supine CT angles are smaller; a similar difference has been reported between standing and supine radiographs [72] because spinal geometry changes significantly between the two positions due to the effect of gravity. Supine CT curve measurements are valuable in biomechanical modeling of scoliosis to give a “zero load” configuration for the spine, which can be used as a starting point for numerical simulations.

Not only can the tortuosity metrics, *M* and *K*, deliver three-dimensional indices of scoliotic spine deformity, they can be used in a fully automated computer measurement system without the need for manual selection of points by the operator.

### Osteoarthritis

**Figure 8:**
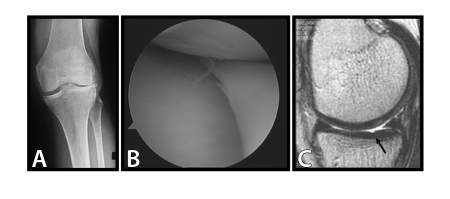
Images of a joint using (a) x-ray (b) arthroscopy and (c) MRI.

Osteoarthritis (OA) is a progressive debilitating disease that results from degradation of the cartilage matrix that provides a low friction surface covering the ends of bones in joints [65]. Degraded cartilage is difficult to distinguish from healthy tissue with current imaging methods until degradation is well-advanced (Figure 8).

**Figure 9:**
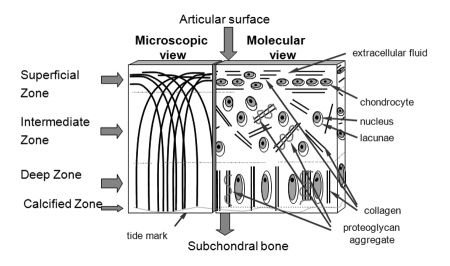
Cartilage microstructure.

The initial stages of OA involve changes in water and proteoglycan content and in the orientation of the collagen fibre bundles in the surface of the cartilage (Figure 9). Recently it has been shown that the collagen fibres restrict the diffusion of water, which can be monitored using diffusion MRI [73].

**Figure 10:**
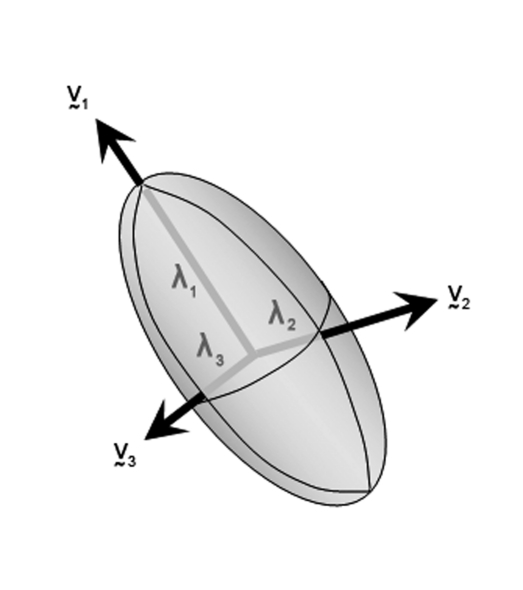
The diffusion ellipsoid is characterised by 3 eigenvectors, v_1,_ v_2_ and v_3_, and 3 eigenvalues λ_1_, λ_2_ and λ_3_.

Diffusion MRI, using a pair of de-phasing and re-phasing gradient pulses with a spin echo MRI sequence [74, 75], characterises these changes by using water diffusion properties as a probe. Diffusion MRI based on a tensor model of the diffusion anisotropy is known as diffusion tensor imaging (DTI). The diffusion tensor can be represented as an ellipsoid, defined by three eigenvectors and three eigenvalues (Figure 10).

**Figure 11:**
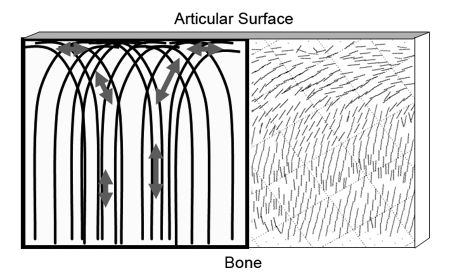
Orientation of collagen fibre bundles in normal cartilage in the form of ‘arcades’ shown schematically at left and a corresponding diffusion tensor image. The projections of the principal eigenvectors are shown as a quiver plot (at right). (After [76]).

The principal eigenvector (viz. the principal direction of diffusion) can be represented by a “quiver” plot, where each quiver represents the projection of the principal diffusion eigenvector on to the image plane (Figure 11). The autocorrelation function (ACF) of the quiver directions, in the articular surface and perpendicular to it, enables a determination of the sizes of the characteristic correlation distances.

**Figure 12:**
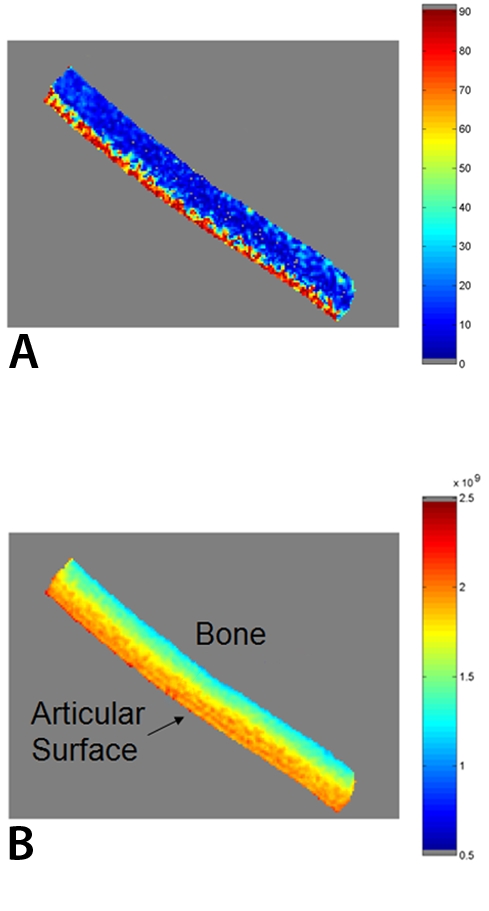
(a) Average orientation of principal eigenvector and (b) maximum diffusion eigenvalues (after [77]).

Alternately the orientation of the principal eigenvector (with respect to the normal articular surface) can be mapped using a colour scale (Figure 12a), as can the maximum (or mean) diffusivity as determined by the principal eigenvalues (Figure 12b). The orientation angles from DTI correlate well with data from polarised light microscopy, PLM [76].

Experiments aimed at better understanding the mechanisms involved in cartilage degradation will continue. Early detection of these changes, when they may still be reversible, is key to the development of new approaches to treatment.
